# 我如何治疗获得性凝血因子缺乏症

**DOI:** 10.3760/cma.j.cn121090-20250905-00414

**Published:** 2025-11

**Authors:** 磊 张, 文静 谷

**Affiliations:** 1 中国医学科学院血液病医院（中国医学科学院血液学研究所），血液与健康全国重点实验室，国家血液系统疾病临床医学研究中心，细胞生态海河实验室，天津 300020 State Key Laboratory of Experimental Hematology, National Clinical Research Center for Blood Diseases, Haihe Laboratory of Cell Ecosystem, Institute of Hematology & Blood Diseases Hospital, Chinese Academy of Medical Sciences & Peking Union Medical College, Tianjin 300020, China; 2 天津医学健康研究院，天津 301600 Tianjin Institutes of Health Science, Tianjin 301600, China

## Abstract

获得性凝血因子缺乏症是一组病因复杂（包括凝血因子抑制物、肝病、弥散性血管内凝血等）的出血性疾病，其中获得性凝血因子抑制物罕见且诊断难度大。临床管理的关键在于早期识别出血倾向、个体化治疗及动态监测。国内针对获得性血友病A已有相对成熟的治疗规范，而其他罕见凝血因子抑制物的临床报道有限。治疗原则需兼顾止血及抑制物清除，对于部分难治性病例可考虑新型疗法。本文结合典型病例，旨在为获得性凝血因子缺乏症的规范化治疗提供实践指导。

## Abstract (en)

Acquired clotting factor deficiency encompasses bleeding disorders with diverse causes, including clotting factor inhibitors, liver disease, and disseminated intravascular coagulation. Among these, acquired clotting factor inhibitors are particularly rare and diagnostically challenging. Clinical management focuses on early recognition of bleeding, individualized therapy, and dynamic monitoring. In China, standardized protocols exist for acquired hemophilia A, while reports on other rare inhibitors are limited. Treatment strategies target both hemostasis and inhibitor eradication, with novel therapies for refractory cases. This article integrates clinical cases to provide practical guidance for standardized management.

获得性凝血因子缺乏症是一组由多种病因引起的获得性出血性疾病，其发病机制涉及凝血因子合成减少（如肝脏疾病）、消耗增加［如弥散性血管内凝血（DIC）］或自身抗体介导的抑制物形成（图1）。这类疾病不同于遗传性凝血障碍，其显著特征为既往无出血史的患者突然出现严重自发性出血。其中，获得性凝血因子抑制物虽然罕见，但其临床表现复杂、易导致严重出血且诊断难度大，尤其需要重点识别。获得性凝血因子抑制物的发病率约为1.5/100万人年，其中以存在抗凝血因子Ⅷ（FⅧ）抗体的获得性血友病A（acquired hemophilia A，AHA）最常见，占所有病例的80％～90％；其次是抗FⅤ抗体和抗血管性血友病因子（vWF）抗体（5％～10％）；其他凝血因子如FⅠ、FⅡ、FⅦ、FⅨ、FⅩ、FⅪ和FXIII等抑制物则更为罕见，总发病率不足5％[1]–[3]。本文将介绍1例AHA典型案例，并结合国内外研究进展，分享笔者对于各类获得性凝血因子缺乏症的治疗经验，供临床医师参考。

**图1:**
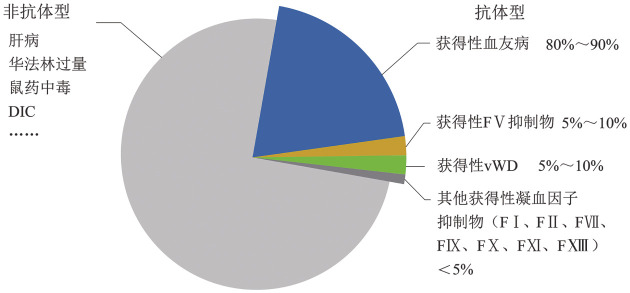
获得性凝血因子缺乏症的分类 **注** DIC：弥散性血管内凝血；vWD：血管性血友病

患者，男，89岁，体重58 kg，主因“乏力1个月，双上肢肿痛10 d”入院。既往有高血压、胃炎病史，无出血性疾病家族史。入院后完善检查，HGB 52 g/L；血清铁1.65 µmol/L，铁饱和度0.03，铁蛋白28.1 ng/ml；凝血功能：活化部分凝血活酶时间（APTT）62.6 s，凝血酶原时间（PT）14.3 s，D-二聚体2.59 mg/L FEU，纤维蛋白降解产物（FDP）8.6 µg/ml；APTT纠正试验：即刻纠正44.9 s，2 h纠正50.5 s；FⅧ活性4.4％，抗FⅧ抗体16.46 BU/ml；狼疮抗凝物（LA）、血小板功能、肿瘤标志物、风湿免疫等指标未见异常。原发病诊断为特发性AHA，同时合并缺铁性贫血。予甲泼尼龙45 mg/d清除抑制物，并予输注悬浮红细胞、抑酸、补铁等对症支持治疗。甲泼尼龙输注4 d后，患者因个人原因要求出院，院外未遵医嘱继续治疗。出院后2周，患者双上肢剧烈活动后肿痛加重，并新发右侧大腿肿痛伴大片皮肤瘀斑，返院治疗。复查HGB 48 g/L，FⅧ活性11.3％，抗FⅧ抗体32.45 BU/ml。立即予输注红细胞纠正贫血，加用重组活化凝血因子Ⅶ（rFⅦa）5 mg/次，每次间隔3 h止血，同时甲泼尼龙45 mg/d联合环磷酰胺0.2 g隔日1次清除抑制物治疗。rFⅦa应用5次后，因经济原因改为凝血酶原复合物（PCC）3 000 IU/d，共输注6 d，四肢血肿较前吸收、疼痛好转，之后停用PCC。患者经甲泼尼龙及环磷酰胺治疗8周后复查FⅧ活性正常，抑制物消失。持续随访，病情稳定无复发。

一、AHA治疗

约半数AHA病例继发于自身免疫性疾病、恶性肿瘤、妊娠或药物等因素，其余患者则无明确诱因。本病的典型表现为严重的皮肤及肌肉软组织出血，若治疗不及时可导致严重并发症甚至死亡。治疗应遵循以下原则：控制急性出血、尽早清除自身抗体以及消除潜在诱因[1]。

（一）止血治疗

一旦确诊AHA，应尽量推迟所有手术、侵入性诊疗操作等至抗体清除后再进行。紧急情况下手术必须在具备丰富经验的医疗中心开展，并确保采取充分的止血防护措施。此外，应避免肌肉注射，并尽量减少静脉穿刺或置管操作，操作需由有经验的医务人员进行。

1. 首选止血治疗：国外指南中推荐的AHA首选止血治疗方案包括旁路制剂rFⅦa、活化凝血酶原复合物（APCC）和重组猪FⅧ（rpFⅧ）[4]。因APCC和rpFⅧ在国内未上市，国内临床主要使用rFⅦa和PCC。rFⅦa的标准用法为90 µg/kg静脉推注，每2～3小时重复给药直至止血。文献报道，其止血有效率超过90％，但需注意潜在血栓风险，EACH2研究显示，rFⅦa血栓事件发生率为2.9％[5]。若无法使用rFⅦa，建议使用PCC 50 IU/kg静脉输注，每8～12小时1次，每日最大剂量不超过150 IU/kg。PCC止血效果较rFⅦa差，且血栓发生率更高。我国CRAE研究报道，rFⅦa和PCC的中位止血时间分别是5 h和72 h，血栓事件发生率分别为0和8.8％[6]。各地区可根据药物可及性和经济成本灵活选择止血治疗方案。

2. 其他止血治疗：其他治疗包括人源性FⅧ及抗纤溶药物，建议仅在旁路制剂无法获取或疗效不佳时使用。人源性FⅧ适用于抗体滴度较低（<5 BU/ml）且具有一定残存FⅧ活性的患者，以短暂提升FⅧ活性，发挥止血作用。首次给予高剂量FⅧ（50～100 IU/kg），在输注后检测FⅧ增量回收率以确定FⅧ活性上升情况。抗纤溶药物（如氨甲环酸、氨基己酸）作为AHA的辅助止血药物，禁用于泌尿道出血的患者。抗纤溶药物应尽量避免与PCC同时使用，建议间隔6 h以上，以降低血栓风险。对于黏膜出血，可局部应用抗纤溶药物替代治疗。

艾美赛珠单抗是一种人源化双特异性单克隆抗体，通过特异性结合FⅨa和FⅩ模拟FⅧ的止血功能，该药最初在先天性血友病A患者中积累了丰富的使用经验。近年来的临床研究证实，其在AHA中同样具有良好的预防出血效果，可显著减少旁路制剂的使用需求，尤其对于基础情况较差的患者，为延迟启动免疫抑制治疗（IST）提供了时间窗（12周内）[7]。艾美赛珠单抗在产后AHA患者中更具应用价值，因这类患者的FⅧ抑制物常可自发消退或对糖皮质激素单药敏感。但大部分患者仍需早期启动IST以实现疾病缓解。

（二）清除抑制物治疗

建议所有患者确诊后，如无禁忌证尽早启动IST，尤其是对于出现严重出血（包括肌肉、腹膜后、颅内及脏器出血）的患者，无论抑制物滴度及残留FⅧ活性如何，都应尽快启动IST以获得疾病完全缓解（CR）（定义为FⅧ活性恢复正常、抑制物转阴且停用免疫抑制剂后无复发）。一线IST方案可选择：①糖皮质激素单药；②糖皮质激素联合环磷酰胺；③糖皮质激素联合利妥昔单抗[8]。治疗方案的选择需基于个体化评估，充分权衡疗效与安全性。对于高龄、基础合并症多的体弱患者，应谨慎评估IST的时机和强度。治疗期间密切监测可能出现的不良反应，如骨髓抑制、感染及糖皮质激素相关不良反应等。

1. 糖皮质激素单药：根据多项研究数据，糖皮质激素单药治疗AHA的CR率为35.2％～76％[5]–[6],[9]–[11]，其缓解率受基线FⅧ活性及抗FⅧ抗体滴度影响。基于GTH研究结果，推荐糖皮质激素单药治疗基线FⅧ活性≥1 IU/dl且抑制物滴度≤20 BU/ml的患者[12]。标准方案为泼尼松1 mg·kg^−1^·d^−1^，疗程4～6周，随后逐渐减量至停药。若FⅧ活性和抑制物滴度持续改善可延长观察期，若治疗3周仍无明显应答，应升级为含环磷酰胺或利妥昔单抗的联合治疗方案。

2. 糖皮质激素联合环磷酰胺或利妥昔单抗：多数研究证实，糖皮质激素联合环磷酰胺或利妥昔单抗的治疗方案较糖皮质激素单药治疗可获得更高的CR率[5]–[6],[9]–[10]，因此建议基线FⅧ活性<1 IU/dl或抑制物滴度>20 BU/ml的患者首选糖皮质激素联合环磷酰胺或利妥昔单抗治疗[12]。环磷酰胺标准给药方案为1.5～2 mg·kg^−1^·d^−1^或200 mg隔日1次，静脉或口服，疗程一般不超过6周。利妥昔单抗推荐给药方案为375 mg/m^2^每周1次，最多4次；或100 mg每周1次，共4次。两种联合治疗方案的疗效对比详见表1。既往AHA的IST方案对比仅限于观察性研究，近年来随着国内外随机对照临床研究的开展，已证实采用单次给药或每周1次（共4次）的利妥昔单抗联合方案，临床疗效不劣于环磷酰胺联合方案。对于年轻育龄期患者，一线选择推荐糖皮质激素联合利妥昔单抗方案[13]–[14]。若一种免疫抑制剂联合方案治疗3～4周无反应，可换用另一种未使用过的免疫抑制剂。

**表1:** 已发表研究中不同免疫抑制方案治疗获得性血友病A的疗效

作者	糖皮质激素单药	糖皮质激素联合环磷酰胺	糖皮质激素联合利妥昔单抗
Collins等[5]	CR率58.0％，达CR时间34 d，复发率18％	CR率80.0％，达CR时间32 d，复发率12％	CR率64.0％，达CR时间46 d，复发率0
Sun等[6]	CR率62.2％，达CR时间69 d，复发率21.7％	CR率87.5％，达CR时间62 d，复发率5.4％	CR率90.9％，达CR时间47 d，复发率5％
Mingot-Castellano等[9]	CR率68.2％，达CR时间30 d	CR率88.5％，达CR时间53 d	CR率87.5％，达CR时间42 d
Schep等[10]	CR率35.2％，达CR时间48 d，复发率25.8％	CR率80.0％，达CR时间75 d，复发率10％	CR率66.7％，达CR时间44 d，复发率25％
Collins等[11]	CR率76.0％，达CR时间49 d	CR率76.0％，达CR时间48 d	–
Wang等[13]	–	CR率68.8％，达CR时间36 d，复发率6.3％	CR率77.4％，达CR时间28 d，复发率6.5％
Lévesque等[14]	–	CR率67.2％，达CR时间46 d	CR率62.0％，达CR时间48 d

**注** CR：完全缓解；–：无数据

3. 新型治疗方案：靶向CD38的单克隆抗体达雷妥尤单抗在AHA治疗领域展现出潜力。我国研究者首次将该药物应用于临床实践，在4例对传统IST无效的难治性AHA患者中采用达雷妥尤单抗（每周4～8 mg/kg）联合糖皮质激素的治疗方案，所有患者均获得CR并维持疗效稳定[15]。目前，针对达雷妥尤单抗的探索性研究已在中国启动，有望为AHA治疗开辟新途径。此外，抗CD19嵌合抗原受体T（CAR-T）细胞疗法作为另一种新型治疗策略，在1例IST耐药的AHA患者中展现出显著疗效和安全性。该患者在CAR-T细胞输注后73 d即达到CR，并在6个月随访期内维持稳定CR，未出现治疗相关不良反应及感染并发症[16]。

（三）疗效监测与随访

在IST期间，建议每周检测1次FⅧ活性、抑制物滴度、血常规及生化等指标，以评估疗效并早期识别IST相关不良反应。获得CR后仍需定期复查FⅧ活性：前6个月每月检测1次，6～12个月期间每2～3个月检测1次，第2年起每半年检测1次，2年后可根据具体情况适当延长检测间隔。抑制物定量根据临床需要适时复查。对于未明确病因者，需持续关注可能的病因，尤其是一线及二线治疗均无效或反复复发时，需进一步排查潜在病因并针对性处理。

本病例中的老年AHA患者以多部位皮肤肌肉出血及重度贫血为主要表现，初诊时FⅧ活性低（4.4％），合并高滴度FⅧ抑制物（16.46 BU/ml）。首次治疗患者因依从性差出现严重出血，且抑制物未得到有效清除（32.45 BU/ml），再次入院经及时有效止血（从rFⅦa过渡至PCC）及IST联合治疗方案，最终实现了FⅧ活性恢复及抑制物清除。由本病例引发思考，AHA治疗需强调以下三点：①AHA常合并危及生命的严重出血，需尽早启动止血治疗，止血药物应根据情况灵活选择，且需加强防护，避免引起严重出血；②IST应足量足疗程，避免过早减量或治疗中断，导致疗效降低甚至无效；③AHA早期诊断及全程规范化治疗是改善预后的关键。

二、其他罕见获得性凝血因子抑制物治疗

与AHA较为成熟的诊疗体系相比，其他获得性凝血因子缺乏的治疗经验多来自个案报道或小样本研究。由于本病罕见且异质性高，诊断更易延误，治疗方案也缺乏统一标准。本部分基于现有国际及国内指南共识和本中心临床实践[17]–[19]，根据止血和抑制物清除两大治疗原则进行阐述。其中止血方案应根据抑制物靶点特异性选择（表2），抑制物清除方案应结合出血严重程度和抑制物效价进行个体化调整。

**表2:** 获得性凝血因子抑制物的止血治疗方案

抑制物类型	可选药物	常用剂量
FⅧ	rFⅦa	90 µg/kg，每2～3小时1次
	PCC	50 IU/kg，每8～12小时1次
	hFⅧ	50～100 IU/kg，根据增量回收率调整剂量
FⅠ	纤维蛋白原浓缩物	50～100 mg/kg
	冷沉淀	1 IU/5～10 kg
FⅡ	PCC	25～50 IU/kg每次
FⅤ	血小板	1～2个治疗量，每24～48小时1次
	FFP	15～20 ml/kg
FⅦ	rFⅦa	90 µg/kg，每2～3小时1次
	PCC	25～50 IU/kg每次
FⅨ	rFⅦa	90 µg/kg，每2～3小时1次
	PCC	25～50 IU/kg/次
FⅩ	PCC	25～50 IU/kg每次
FⅪ	rFⅦa	90 µg/kg，每2～3小时1次
	PCC	25～50 IU/kg每次
FXIII	冷沉淀	1 IU/5～10 kg
	FFP	15～20 ml/kg
vWF	DDAVP	0.3 µg/kg每次，输注超过30 min
	vWF浓缩物	30～100 IU/kg
	rFⅦa	90 µg/kg，每2～3小时1次

**注** vWF：血管性血友病因子；rFⅦa：重组活化凝血因子Ⅶ；PCC：凝血酶原复合物；hFⅧ：人源性凝血因子Ⅷ；FFP：新鲜冰冻血浆；DDAVP：去氨加压素

（一）止血治疗

1. 纤维蛋白/纤维蛋白原（FⅠ）缺乏：抗FⅠ自身抗体通过影响纤维蛋白形成、聚合及稳定性导致严重自发性反复出血，多见于系统性红斑狼疮、意义未明单克隆丙种球蛋白血症（MGUS）及骨髓瘤患者。止血治疗首选补充纤维蛋白原浓缩物，次选冷沉淀，而新鲜冰冻血浆（FFP）纤维蛋白原含量低，止血效果有限。

2. 凝血酶/凝血酶原（FⅡ）缺乏：抗FⅡ自身抗体常见于接受牛凝血酶治疗或自身免疫性疾病患者。部分LA可直接结合FⅡ，导致其清除加速或活性受抑，从而引发获得性低凝血酶原血症-狼疮抗凝物综合征（HLAS），临床可表现为严重出血，偶见血栓形成。止血治疗可选择PCC中和抑制物，同时提供部分旁路活性，但需定期监测FⅡ、FⅦ、FⅨ、FⅩ水平。由于rFⅦa无法补充凝血酶原，疗效有限。

3. FⅤ缺乏：FⅤ自身抗体常发生于接受牛凝血酶或抗生素治疗、恶性肿瘤及自身免疫性疾病患者，出血严重程度与FⅤ水平相关。可输注FFP止血，但因FFP中FⅤ浓度有限且易被循环抗体中和，止血效率仅为15％左右。血小板输注反应率更高，可达30％，因血小板富含FⅤ并能靶向释放至出血部位[20]。难治性出血的挽救治疗包括大剂量FFP或旁路制剂。大剂量静脉注射免疫球蛋白（IVIG）可能有效[21]。

4. FⅦ缺乏：获得性FⅦ抑制物与感染、自身免疫性疾病及恶性肿瘤相关，治疗应首先针对原发病。止血措施可选择rFⅦa以及PCC，但需加大剂量以中和抑制物，否则疗效有限。

5. FⅨ和FⅪ缺乏：获得性FⅨ和FⅪ抑制物的报道较为罕见，多为自身免疫性疾病或恶性肿瘤诱发。止血治疗方案与AHA类似，首选rFⅦa，若无效可换用另一种旁路制剂，如PCC。

6. FⅩ缺乏：获得性FⅩ缺乏主要继发于淀粉样变性，其次由FⅩ抑制物导致。淀粉样变性所致FⅩ缺乏可被正常血浆纠正，因其机制为淀粉样物质对FⅩ的物理吸附而非免疫抑制。FⅩ抑制物则多见于合并感染或恶性肿瘤患者。治疗应首先针对原发病，若发生出血，可用PCC止血，但需密切监测凝血因子水平以避免血栓风险。不推荐rFⅦa，其止血作用需依赖FⅩ激活，因此疗效有限。

7. FXIII缺乏：获得性FXIII抑制物通过抑制凝血酶对FXIII的激活及阻断FXIII与纤维蛋白结合位点影响凝血功能。临床多表现为严重出血，如关节血肿、月经增多和颅内出血等。止血治疗首选大剂量FXIII浓缩物，因国内暂无FXIII浓缩物制品，可用冷沉淀替代。冷沉淀中FXIII含量有限，但显著高于FFP，需密切观察出血症状，输注后可复查FXIII活性，以明确输注疗效。

8. vWF缺乏：获得性血管性血友病（AVWD）多继发于心血管疾病、骨髓增殖性肿瘤（MPN）、淋巴增殖性疾病（LPD）及自身免疫性疾病等，以原发病治疗为基础，止血治疗应根据不同疾病特点而定。由物理因素（如心血管疾病）或循环吸附（如MPN、部分LPD）引起的vWF减少，可选用去氨加压素（DDAVP）、vWF浓缩物替代治疗；由vWF中和抗体所致vWF活性重度减低（如自身免疫性疾病、部分LPD），vWF替代治疗无效，可选择旁路途径rFⅦa止血。对于IgG型MGUS相关AVWD，可采用IVIG 1 g·kg^−1^·d^−1^×2 d提高vWF活性以达到止血目的，疗效可维持约3周[22]。

（二）清除抑制物治疗

对于无出血症状的获得性FⅤ、FⅨ、FⅪ缺乏患者可考虑观察随访，部分抑制物可能呈一过性升高或持续存在而无出血表现。有出血症状的患者，确诊后应尽早启动IST，推荐参照AHA的IST方案[18]。在各类罕见获得性凝血因子缺乏症中，IST的CR率存在明显差异。笔者统计了本中心收治的17例获得性FⅤ缺乏症患者，30％接受糖皮质激素单药治疗，60％采用糖皮质激素联合环磷酰胺或利妥昔单抗方案，总体CR率达76.5％。相比之下，获得性FⅨ和FXIII缺乏的CR率较低，为50％左右。FⅩ缺乏患者无论接受IST还是化疗方案，其CR率均不足20％[23]。血浆置换或免疫吸附治疗可增加抑制物清除速度。临床中非中和抗体的检测，传统Bethesda法敏感性较差，可采用ELISA、免疫沉淀等免疫学方法或清除动力学分析综合评估。

对于部分难治性病例，若传统IST方案无效，可尝试新型治疗方案。本中心曾收治1例骨髓增生异常综合征确诊后8个月出现皮肤肌肉出血的患者，实验室检查发现FXIII活性11.5％，FXIII抑制物1.45 BU/ml。患者经冷沉淀、FFP输注止血，甲泼尼龙40 mg/d（体重约50 kg）联合达雷妥尤单抗400 mg/周（共5周）清除抑制物治疗后，FXIII活性恢复正常，抑制物转阴。凝血异常纠正后患者顺利完成6个疗程去甲基化治疗，随访至今FXIII活性正常，未再出现异常出血。

三、非抗体介导的凝血因子缺乏症治疗

（一）肝病相关凝血因子缺乏

肝病患者因肝脏合成功能受损，FⅡ、FⅤ、FⅦ、FⅨ、FⅩ、FⅪ生成减少，同时维生素K依赖性凝血因子（FⅡ、FⅦ、FⅨ、FⅩ）激活受限。此外，肝病患者常合并脾功能亢进所致血小板减少、纤维蛋白溶解亢进及纤维蛋白原水平减低，多重机制共同加重凝血紊乱。临床表现为显著的出血倾向，包括皮肤黏膜出血、消化道出血和术后创面出血，严重者可进展至消耗性凝血障碍甚至DIC。治疗策略以积极治疗肝病为核心，对于慢性病程可适当补充维生素K，但应避免肌肉注射。急性出血或高风险侵入性操作前可输注FFP或PCC补充凝血因子，输注纤维蛋白原浓缩物以维持血清纤维蛋白原浓度>120 mg/dl。非紧急情况下血小板减少可使用血小板生成素受体激动剂替代血小板输注以避免循环负荷过重，抗纤溶药物作为止血的辅助治疗，应用时需密切监测以防止血栓形成[24]。

（二）维生素K依赖性凝血因子缺乏（VKDFD）

VKDFD是由于维生素K摄取不足、吸收障碍（胆道疾病、慢性腹泻）或利用受阻（华法林过量、鼠药中毒）所致的FⅡ、FⅦ、FⅨ、FⅩ、蛋白C（PC）、蛋白S（PS）活性减低。维生素K缺乏可影响凝血因子羧化及其与钙离子结合，进而影响凝血级联反应。临床表现以出血倾向为主，轻者表现为皮肤黏膜出血，重者可出现消化道出血、颅内出血等危及生命的出血事件。治疗上以去除病因为主，同时补充维生素K，急性出血时首选静脉输注，给药速度不高于1 mg/min。严重出血时联合输注FFP或PCC以快速补充凝血因子。对于慢性吸收障碍的患者，需长期口服补充维生素K。

（三）DIC相关凝血因子缺乏

DIC是由严重感染、恶性肿瘤或病理产科等原发疾病触发的凝血系统过度活化，微血管内广泛血栓形成，进而导致凝血因子（FⅠ、FⅡ、FⅤ、FⅧ、FⅩ等）和血小板大量消耗的病理过程。DIC患者常合并不同程度的出血表现，治疗的关键在于寻找诱因并及时有效地治疗原发病，多数病例在原发病有效治疗后凝血异常可逐步恢复。对于活动性出血或拟行侵入性操作的患者，需输注血制品替代治疗，包括FFP、冷沉淀、纤维蛋白原浓缩物及血小板等，并根据临床分期酌情选择肝素抗凝或抗纤溶治疗[25]。

获得性凝血因子缺乏症虽相对少见，但其病因复杂且可能危及生命，临床需高度重视并及时明确诊断。确诊后，止血治疗不应局限于单纯补充缺失的凝血因子，而应深入分析潜在病因并实施针对性干预：由抑制物介导的患者需行IST以清除抑制物，肝病所致患者应重点改善肝功能以促进凝血因子合成，DIC患者需控制基础疾病以减少凝血因子消耗，药物或毒物相关患者应及时去除诱因。止血治疗为当务之急，病因治疗是改善预后的关键。
